# The visualization of stress in clinical training: A study of nursing students' perceptions

**DOI:** 10.1002/nop2.629

**Published:** 2020-09-23

**Authors:** Waddah Mohammad D'emeh, Mohammed Ibrahim Yacoub

**Affiliations:** ^1^ Community Health Nursing Department School of Nursing The University of Jordan Amman Jordan; ^2^ Department of Clinical Nursing School of Nursing The University of Jordan Amman Jordan

**Keywords:** clinical training, nursing students, stress level, stress type, Saudi Arabia

## Abstract

**Aim:**

To determine the perceived level and types of stress experienced by nursing students during clinical training.

**Design:**

A descriptive correlational cross‐sectional design.

**Methods:**

A convenience sample of 238 nursing students was recruited. Level and types of stress were measured by Perceived Stress Scale. The data were collected in February 2020.

**Results:**

The mean score of perceived stress was 2.58 (*SD* 0.92). Different sources of stressors perceived by students were identified in current study. The highest type of stressors perceived by students was stress from taking care of patients (mean = 2.81, *SD* 1.13). In addition, junior students perceived higher level of stress than senior students and female students experienced a higher level of stress than male counterparts. Students who have been supervised by clinical instructors whom their “primary” language is English scored a higher level of stress.

## 1 INTRODUCTION

Nursing education is considered one of the most stressful program compared with other academic disciplines and university majors (Reeve et al., 2013). Lazarus and Folkman (1987) defined stress as “situation in which internal demands, external demands, or both are appraised as taxing or exceeding the adaptive or coping resources of an individual or group” (p. 19). Nursing education programs consist of theoretical education and clinical education. Nursing students experience a day life stress during their education, particularly during clinical training (Labrague, McEnroe‐Petitte, De Los Santos, et al., 2018). Clinical training accounts for 40%–50% of each program's credit hours. Clinical training is an opportunity for students to apply what they have learned in classrooms and develop their capabilities. Bastable (2014) defines clinical teaching as the mode where students translate theoretical knowledge into the learning of a variety of skills required to give patient‐centred care.

Two types of sources of stress for nursing students have been documented in the literature. They are stresses induced by academic and those induced by clinical training (Labrague et al., 2017). Clinical stressors include uncertainty about the unknown, fear of making mistakes (Pulido‐Martos et al., 2012), taking care of patients, nursing staff and nurse educators (Al‐Gamal et al., 2018), fear of failure (Sharif & Masoumi, 2005), lack of knowledge or nursing skills, unfamiliarity with patient history, diagnosis or treatment (Sheu et al., 2002), peers, daily life and university environment (Shaban et al., 2012), learning new responsibilities (Seyedfatemi et al., 2007), assignments and workload, caring for dying or terminally ill patients and incongruence between class instructions and clinical instructions (Hamaideh et al., 2017; Zhao et al., 2015).

Tolerated, minimal amount of stress could motivate students to enhance further success. However, higher level of stress can adversely influence students. Unresolved stress has been documented to affect student's overall health and quality of life (Labrague, McEnroe‐Petitte, Papathanasiou, et al., 2018). For example, stress due to academic demands can cause anxiety, sleep disturbances, substance use, lack of concentration (Pascoe et al., 2020), poor social life and depression (Yamashita et al., 2012). Unfortunately, 17.1% of male and 20.9% of female undergraduate college students felt so depressed that it was difficult to function in last 12 months of college matriculation (American College Health Association, 2019). Other physical symptoms include high blood pressure, cardiac diseases, gastrointestinal upset and immune deficiency diseases (Lee et al., 2007). Furthermore, stress affects student's decision‐making skills, which in turn affect the quality of care provided to patients (Chan et al., 2009), and therefore, it could lead to academic burnout (Lin & Huang, 2013) and unhealthy stress‐coping behaviours (Ab Latif & Mat Nor, 2019).

## 2 BACKGROUND

Multiple factors contribute to nursing students' stress in clinical setting in Saudi Arabia. First, attitude of nursing staff and other healthcare professionals, particularly non‐Saudi staff (Albloushi, Ferguson, et al., 2019). Second, students' lack of English skills which interfere with their communication with staff as a large proportion of nurses who are working in clinical settings are expatriate nurses and they speak English as a primary language. Although the nursing' program curriculum is English‐based, English language barrier is considered significant. This is true not only in relation to communication with staff, but also regarding reading and understanding hospital documents and patient's files. Therefore, these aforementioned factors affect students' confidence and self‐esteem (Alhazmi & Windsor, 2013). Third, unsupportive clinical educators who were unable to create positive clinical environment or help students to feel safe, secured and confident in providing care for patients. Most of faculty members and clinical instructors are expatriate whom speak English, some of them lack clinical experience (Albloushi, Ferguson, et al., 2019). Fourth, students experience cultural challenges such as cross‐gender interaction, family disagreement, gender desegregation, negative image of nursing and perception about a stressful profession (Aboshaiqah, 2016). Fifth, challenging work and organizational environment: low pay, no incentives, overload and treating nurses as inferior to other medical profession (Albloushi, Alghamdi, et al., 2019). Sixth, inadequate preparation; usually, clinical training starts early in nursing program where students are not well equipped and prepared to provide adequate care for patients (Alhazmi & Windsor, 2013). Finally, other factor reported and represent a concern, lack of governmental financial support or scholarships, incompatible curriculum with healthcare system development, and way of choosing nursing as a profession (Albloushi, Alghamdi, et al., 2019).

Out of previously published studies in Saudi Arabia and up to authors' knowledge, one study has recruited students from private nursing colleges. Hamaideh et al. (2017) used Perceived Stress Scale (PSS) and Coping Behavior Inventory to describe levels, types of stress and coping behaviours of nursing students during their clinical training. Students scored moderate level of stress using PSS (mean = 1.46, *SD* 0.66). Assignments and workload, teacher and nursing staff, and the environment were the main sources of stress reported by students.

Current study intended to provide better understanding about the level and sources of stress experienced by nursing students' during their clinical training. Additionally, results from this study could help nursing educators in designing stress‐management strategies to lessen students' stress and provide students with supportive and caring clinical environment. Moreover, understanding of students' stressors will help in identifying strategies that can help students reduce their stress, enhance students' success, improve their motivation for learning and retention and attract prospective nurses to join the profession (Clark et al., 2014). The aim of this study was to determine perceived level of stress and types of stress reported by Saudi nursing students during clinical training. This study answered the following questions: (1) What is the perceived level of stress reported by Saudi nursing students during their clinical training? (2) What are the types of stressors reported by Saudi nursing students during their clinical training? (3) What is the relationship between perceived level of stress and selected demographic factors?

## 3 METHODS

### 3.1 Design

A descriptive, correlational cross‐sectional design was employed in this study.

### 3.2 Setting and sample

Data were collected from three different private nursing schools in universities located in Riyadh city, Saudi Arabia in February 2020. All enrolled students in the selected universities were invited to participate in the study. A total of 280 nursing students were invited to participate. A convenience sample of 238 students was recruited and composed the final study sample. Each student was registered for at least one clinical course. Nursing program in the selected universities consists of 4 years (8 levels) of study and one‐year internship. Students spent around 1,800 contact hours during their clinical study period. Students begin their clinical training in level 4 spending two days per week in clinical areas (hospitals) for about 8 hours each day, each clinical course lasts for 12 weeks. Students were supervised in clinical setting by clinical instructor who hold Bachelor's or Master degree in Nursing.

### 3.3 Instrumentations

The instrumentation package included consent form, demographic sheet and Perceived Stress Scale. The demographic sheet includes the following factors: age, gender, education level (4–8), Grade Point Average (GPA out of 5), marital status, way of choosing nursing, financial status, employability, type of school admission, clinical instructor's gender, clinical instructor's “primary” language and number of registered hours. The Perceived Stress Scale was used to measure level and type of stress. The PSS was originally developed by Sheu et al. (1997). The scale consists of 29 items using a five‐point Likert scale, the responses range from “never” to “always” and are scored from 0–4, total score ranges from 0–116. The higher score means higher level of stress while the lower score means lower level of stress. The scale included 6 subscales: stress from taking care of patients (8 items), stress from teachers and nursing personnel (6 items), stress from assignment and workload (5 items), stress from peers and daily life (4 items), stress from lack of professional knowledge and skills (3 items) and stress from the clinical environment (3 items). The scale and subscales showed acceptable reliability and validity (Chan et al., 2009; Sheu et al., 2002). A translated version of the scale (Al‐Gamal et al., 2018) was used in the current study.

### 3.4 Ethical considerations

Ethical approval was obtained from institutional review board (IRB) and ethical committees in selected universities. All participants were informed about the purpose and procedure of the study, voluntary nature of participation, right to refuse or withdraw from participation at any time without any penalties. Anonymity was assured through coding of questionnaires. Also, confidentiality was maintained through restriction of access to collected data.

### 3.5 Data collection procedure

Data collected at the beginning of clinical rotation to eliminate any confounding factors. The principle researcher approached students in classrooms, explained to them the study purpose and procedure and invited them to participate in the study. Students who agreed to participate were instructed to sign the consent form and completed study questionnaire and to returned it back to sealed box provided by the researcher. The time needed to complete the questionnaires was approximately 20 minutes.

### 3.6 Statistical analysis

Data were managed using IBM SPSS, version 22. Descriptive statistical measures were used to describe the sample and the items of the scale. One‐way ANOVA was used, followed by Tukey's post hoc test to examine the difference by schools, education level, gender, financial status, employability, clinical instructor “primary” language and PSS subscales. Significance level was set at *p* < .05.

## 4 RESULTS

### 4.1 Characteristics of the study sample

Response rate was 85% (238/280). Characteristics of the sample were summarized in Table 1. Most participants (79.4%) were female, 66% of them worked full time and 87.4% pay for their study.

**TABLE 1:** Characteristics of the sample

Variable	Frequency	Percentage	Mean	*SD*
Age			22.86	2.9
GPA out of 5			2.96	1.39
Number of registered hours			15.23	2.17
Gender				
Female	189	79.4		
Male	49	20.6		
Marital status				
Single	198	83.2		
Married	34	14.3		
Divorce/separated	6	2.5		
Education level				
Level 4	39	16.4		
Level 5	44	18.5		
Level 6	32	13.4		
Level 7	69	29		
Level 8	54	22.7		
Employability				
Full time	66	27.7		
Part time	48	20.2		
Unemployed	124	52.1		
Way of choosing nursing				
Self‐selection	177	74.4		
Family selection	61	25.6		
Financial status				
Self‐funded	208	87.4		
Assistantship	30	12.6		
Type of school admission				
Secondary school	162	68.1		
Bridging program	76	31.9		
Clinical instructor gender				
Female	21	72.4		
Male	8	27.6		
Clinical instructor “primary” language				
Arabic	19	65.5		
English	10	34.5		

Abbreviation: *SD*, Standard deviation.

### 4.2 Levels and types of stress

The level of stress perceived by students ranged from 22–106. The mean of perceived stress was 2.58 (*SD* 0.92). Types of stressors perceived by students were (1) stress from taking care of patients (mean = 2.81, *SD* 1.13), followed by (2) stress from teachers and nursing staff (mean = 2.55, *SD* 1.08), (3) stress from lack of professional knowledge and skills (mean = 2.39, *SD* 1.01), (4) stress from assignments and workload (mean = 2.29, *SD* 0.95), (5) stress from peers and daily life (mean = 2.02, *SD* 0.96) and (6) stress from the environment (mean = 1.88, *SD* 0.91). The highest stressful event students experienced was “Do not know how to discuss patients' illness with teachers or medical and nursing personnel” (mean = 3.02, *SD* 0.91), followed by “Lack of experience and ability in providing nursing care and in making judgments” (mean = 2.88, *SD* 1.09). The lowest stressful event students experienced was “Experience competition from peers in school and clinical practice” (mean = 1.67, *SD* 0.86) (Table 2).

**TABLE 2:** Stressors perceived by nursing students in clinical training

Stressors	Subscale rank	Mean	*SD*
1. Stress from taking care of patients			
Lack of experience and ability in providing nursing care and in making judgments	1	2.88	1.09
Do not know how to help patients with physio‐psycho‐social problems		2.76	1.02
Unable to reach one's expectations		2.31	1.03
Unable to provide appropriate responses to doctors', teachers', and patients' questions		2.46	1.02
Worry about not being trusted or accepted by patients and/or patients' family		2.68	1.11
Unable to provide patients with good nursing care		2.29	1.01
Do not know how to communicate with patients		2.33	1.06
Experience difficulties in changing from the role of a student to that of a nurse		2.18	1.21
2. Stress from teachers and nursing staff			
Experience discrepancy between theory and practice	2	2.81	0.94
Do not know how to discuss patients' illness with teachers or medical and nursing personnel		3.02	0.91
Feel stressed that teacher's instruction is different from one's expectations		2.27	1.21
Medical personnel lack empathy and are not willing to help		2.49	1.12
Feel that teachers do not give fair evaluation on students		2.32	0.99
Lack of care and guidance from teachers		2.40	1.03
3. Stress from lack of professional knowledge and skills			
Unfamiliar with medical history and terms.	3	2.42	0.98
Unfamiliar with professional nursing skills.		2.38	0.96
Unfamiliar with patients' diagnoses and treatments.		2.51	1.05
4. Stress from assignments and workload			
Worry about bad grades	4	2.53	1.06
Experience pressure from the nature and quality of clinical practice		2.32	1.12
Feel that one's performance does not meet teachers' expectations.		2.10	1.08
Feel that the requirements of clinical practice exceed one's physical and emotional endurance.		2.22	0.93
Feel that dull and inflexible clinical practice affects one's family and social life		2.09	0.88
5. Stress from peers and daily life			
Experience competition from peers in school and clinical practice	5	1.67	0.86
Feel pressure from teachers who evaluate students' performance by comparison		2.01	0.96
Feel that clinical practice affects one's involvement in extracurricular activities		1.85	1.00
Cannot get along with other peers in the group		1.69	0.97
6. Stress from the environment			
Feel stressed in the hospital environment where clinical practice takes place	6	1.98	1.07
Unfamiliar with the ward facilities		1.76	1.01
Feel stressed from the rapid change in patient's condition		2.06	0.93

Abbreviation: *SD*: Standard deviation.

Differences in level and sources of stress among schools were shown in Table 3. The analyses showed significant results as cause of stress from teachers and nursing staff (*p* < .001) and from assignments and workload (*p* < .01). A statistically significant relationship was found between subscales of PSS and the total score (*p* < .001). The analyses revealed that students in level 4 and level 5 perceived higher level of stress than student from other levels (mean = 81.49, *SD* 22.39), (mean = 77.54, *SD* 20.76). Both groups perceived stress from lack of professional knowledge and skills (*p* < .001), from taking care of patients (*p* < .001), from the environment (*p* < .02). Female students experienced higher stress level than male students in two subscales: Stress from assignments and workload (*p* < .01) and Stress from teachers and nursing staff (*p* < .03). The stress perceived by self‐funded students (mean = 78.21, *SD* 21.99) and full‐time employed students were statistically significant (mean = 79.11, *SD* 20.14). Finally, students who have been supervised by clinical instructor whom their “primary” language is English scored higher level of stress (mean = 80.69, *SD* 21.11). Other demographical factors showed no statistically significant difference.

**TABLE 3:** Differences in nursing students' mean scores of perceived stress

Factor	*N*	Percentage	Perceived Stress Scale (PSS)						
			1. Stress from taking care of patients	2. Stress from teachers and nursing staff	3. Stress from lack of professional knowledge and skills	4. Stress from assignments and workload	5. Stress from peers and daily life	6. Stress from the environment	PSS Total score
			Mean (*SD*)	Mean (*SD*)	Mean (*SD*)	Mean (*SD*)	Mean (*SD*)	Mean (*SD*)	Mean (*SD*)
Schools									
School 1	94	39.5	22.13 (6.08)	19.36 (3.88)	7.89 (4.01)	14.87 (3.29)	7.86 (3.32)	7.15 (3.89)	79.26 (24.47)
School 2	78	32.8	20.19 (4.98)	17.25 (3.07)	6.81 (3.62)	15.21 (2.98)	9.16 (4.08)	7.59 (4.03)	76.21 (22.76)
School 3	66	27.7	23.50 (7.11)	18.96 (2.45)	6.32 (3.55)	13.80 (3.22)	7.36 (3.71)	8.23 (3.79)	78.17 (23.83)
	238	100	*F*_2,235 = 2.31_	*F*_2,235 = 4.98_	*F*_2,235 = 1.69_	*F*_2,235 = 8.26_	*F*_2,235 = 1.97_	*F*_2,235 = 2.04_	*F*_2,235 = 3.57_
			*p* = .15	*p* = .001	*p* = .11	*p* = .01	*p* = .06	*p* = .08	*p* = .001
Education level									
Level 4	39	16.4	23.59 (5.83)	19.88 (4.02)	9.01 (2.78)	13.79 (3.26)	7.93 (3.31)	7.79 (3.19)	81.49 (22.39)
Level 5	44	18.5	21.88 (5.19)	18.21 (3.66)	7.69 (2.86)	14.55 (3.18)	7.66 (3.28)	7.55 (2.59)	77.54 (20.76)
Level 6	32	13.4	19.13 (5.89)	17.51 (3.39)	7.19 (3.01)	14.39 (3.85)	8.12 (3.58)	6.89 (3.51)	79.26 (24.47)
Level 7	69	29	19.01 (4.78)	17.25 (3.01)	6.69 (2.92)	15.01 (3.65)	8.36 (3.598)	7.01 (3.11)	76.21 (22.76)
Level 8	54	22.7	17.82 (4.64)	17.16 (3.50)	6.28 (3.01)	14.06 (2.87)	7.36 (4.01)	6.39 (3.02)	78.17 (23.83)
	238	100	*F*_4,234 = 3.69_	*F*_2,234 = 2.98_	*F*_2,234 = 4.69_	*F*_2,234 = 2.26_	*F*_2,234 = 6.32_	*F*_2,234 = 1.84_	*F*_2,234 = 3.57_
			*p* = .001	*p* = .07	*p* = .00	*p* = .001	*p* = .06	*p* = .02	*p* = .00
Gender									
Female	189	79.4	23.08 (5.96)	20.16 (3.28)	8.09 (3.59)	15.11 (3.69)	8.42 (3.42)	7.15 (3.89)	79.26 (24.47)
Male	49	20.6	20.89 (5.18)	18.95 (2.91)	7.81 (3.62)	14.51 (2.40)	8.16 (3.69)	7.59 (4.03)	76.21 (22.76)
	238	100	*F*_1,233 = 2.81_	*F*_1,233 = 1.68_	*F*_1,233 = 3.51_	*F*_1,233 = 2.23_	*F*_1,233 = 1.97_	*F*_1,233 = 2.04_	*F*_1,233 = 3.57_
			*p* = .09	*p* = .03	*p* = .18	*p* = .01	*p* = .06	*p* = .08	*p* = .00
Financial status									
Self‐funded	208	87.4	21.68 (5.39)	20.11 (3.08)	7.12 (3.26)	13.61 (4.01)	8.23 (3.03)	7.46 (3.22)	78.21 (21.99)
Assistantship	30	12.6	20.16 (5.08)	19.29 (2.32)	6.91 (4.12)	14.81 (3.26)	8.11 (3.21)	7.28 (3.12)	76.56 (21.11)
			*F*_1,233 = 1.77_	*F*_1,233 = 1.98_	*F*_1,233 = 4.18_	*F*_1,233 = 3.74_	*F*_1,233 = 6.39_	*F*_1,233 = 5.14_	*F*_1,233 = 2.25_
			*p* = .00	*p* = .04	*p* = .19	*p* = .02	*p* = .06	*p* = .10	*p* = .001
Employability									
Full time	66	27.7	20.18 (4.91)	19.86 (3.32)	7.81 (2.81)	15.72 (3.16)	8.13 (2.71)	7.41 (3.23)	79.11 (20.14)
Part time	48	20.2	20.67 (4.84)	18.86 (3.17)	7.69 (3.41)	14.61 (3.28)	8.26 (3.29)	7.69 (3.41)	77.78 (21.40)
Unemployed	124	52.1	21.37 (5.33)	18.18 (3.29)	6.92 (3.09)	13.67 (3.02)	8.06 (2.61)	8.18 (3.28)	76.38 (20.62)
			*F*_2,233 = 2.01_	*F*_2,233 = 1.86_	*F*_2,233 = 2.96_	*F*_2,233 = 6.29_	*F*_2,233 = 4.62_	*F*_2,233 = 3.44_	*F*_2,233 = 2.18_
			*p* = .02	*p* = .01	*p* = .11	*p* = .00	*p* = .19	*p* = .24	*p* = .01
Clinical instructor “primary” language									
Arabic	19	65.5	18.89 (5.39)	18.63 (2.78)	7.28 (3.16)	14.53 (2.81)	7.56 (3.13)	7.96 (2.82)	74.85 (20.09)
English	10	34.5	21.06 (5.18)	20.88 (3.12)	7.95 (3.58)	14.91 (3.29)	8.21 (2.74)	7.68 (3.20)	80.69 (21.11)
			*F*_1,233 = 2.16_	*F*_1,233 = 1.83_	*F*_1,233 = 6.39_	*F*_1,233 = 3.37_	*F*_1,233 = 4.29_	*F*_1,233 = 4.14_	*F*_1,233 = 1.69_
			*p* = .00	*p* = .001	*p* = .07	*p* = .05	*p* = .23	*p* = .15	*p* = .001

Abbreviation: *SD*, Standard deviation.

## 5 DISCUSSION

This study aimed to determine the level of and sources of stress among Saudi nursing students during their clinical training. This study provided us with better understanding about level and types of stress experienced by Saudi nursing students. The results of this study suggest that the level of stress among Saudi nursing students is high. Similarly, increased level of stress was reported by other studies (Karaca, 2015). This result should drive the attention of nursing educators to better understand what would cause this magnitude and propose strategies that lessen or eliminate sources of stress.

The findings of this study showed that highest source of stress was from taking care of patients. This is congruent with other studies (Al‐Zayyat & Al‐Gamal, 2014; Chen & Hung, 2014). Alleviating sources of stress could be achieved by designing strategies that prepare students about patients care before they start their clinical rotation through reviewing clinical curriculum that is clinically oriented in schools' laboratories and in high‐fidelity simulation laboratories that provide students with safe and supportive opportunities to train before they deal with actual patients (Turner & McCarthy, 2017). It is noteworthy that nursing schools should provide student with comprehensive clinical orientation program that help them bridging the gap between theory and practice and retain knowledge before their practice. In addition, selection of clinical area should be suited to students' level of knowledge and skilled. Finally, clinical instructors need to be readily available for students and able to assess their stress and work with them on competencies so they are will prepared for clinical settings.

The second highest source of stress reported was from teachers and nursing staff. This finding reported in (Chan et al., 2009). Clinical settings in Saudi Arabia are described as multicultural complex environment. Clinical instructors and health workers are coming from different nationalities and use English as a main language of communication. This result call for an assessment and review of clinical teachers' qualifications and competencies, student–instructor relationship, students–nursing staff relationship and students' workload. It is the responsibility of nursing school administrators to hire qualified clinical instructors that are able to meet program outcomes and be able to implement strategies that enhance open intercommunication techniques between themselves, students and staff nurses. Adopting preceptorship program in clinical areas would be of great benefit to lessen this kind of stress as staff nurses and clinical teacher work together collaboratively to achieve clinical practice desired outcomes through definitive clear plans.

Clinical instructors should communicate to nursing staff and other healthcare team members about the need of students for welcoming, supportive and caring relations during their clinical training. Clear expectations between all parties should be explained from the beginning so unnecessary conflict can be avoided. Another crucial finding of this study was the “primary” language of clinical teacher as a barrier from students' perspectives as students scored higher stress level when their instructor's primary language is English. To some extent, this result was expected since Saudi nursing students and other universities' students are lacking English language skills. Lack of appropriate verbal and written communication contribute to affect students' confidence, self‐esteem and intensify feeling of inadequacy, which ultimately increase students' level of stress. It is the role of university leaders where medical sciences majors offered to mandate an English language skill proficiency courses to prepare future students for such academic programs to ease students' academic and clinical education.

Not a surprising finding was the significant results that related to students' education level. Student from level 4 and level 5 (junior students) reported higher level of stress, which is similarly reported by to other studies (Alsaqri, 2017). An explanation could be that junior students are not fully exposed to vast amount of knowledge or skills and not well prepared and equipped professionally and emotionally in comparison with senior students. The early encounter of junior students with highly technological environment and unfamiliar assignments and hospital work contribute to such results (Khater et al., 2014). Student during this initial period of clinical training is unable to help patients with physio‐psych‐social problems and were limited in their professional knowledge and skills (Sheu et al., 2002). Early identification of source of stress at this level could represent an opportunity to clinical instructors to eliminate or lessen these stressors. As previously mentioned, clinical educator can play an important role in preparing students prior to clinical rotation. Also, instructors and preceptors should be available to students when help is needed. Senior students in our sample are admitted to nursing school through bridging program path, these students were exposed to clinical setting before they joined school and this could also explain their lower level of stress compared with their peers.

Incongruent with other studies, female students who count for around 80% of the sample reported higher stress than male students. This can be attributed to some cultural factors such as cross‐gender interactions, public negative image of nursing and expression of feelings and emotions explicitly. To add, most of stressed female students were full‐time workers and married, which represent an additional responsibility. The finding revealed that students who pay for their study reported a higher level of stress compared with other students. This can be related to the responsibility they feel and the worries they hold about grades and passing program courses in the planned time. It is worthy to mention that government used to provide private university's students with full scholarships that cover tuition and life expenses before year 2016. These scholarships are not offered any more and most of students in private universities “now” are self‐funded. In response to the shortage of Saudi nurses, the government should have plans to attract Saudi prospective nurses to join the profession either by providing partial or full assistantships.

### 5.1 Limitations

This study was conducted among students enrolled in nursing program at private nursing schools only by using a convenient sample. This would limit the generalizability of the study. The study used self‐report questionnaire which make responses subject to participants' personal interpretation. Further research are needed to study the stress per nursing patient or topic not by education level alone. In addition, a qualitative approach would give more insight into the individual experience regarding academic stress.

## 6 CONCLUSION AND IMPLICATIONS

The findings of this study demonstrated a high level of stress among Saudi nursing students in clinical settings. Resolving sources of stress should be carried out through implementing different strategies by nursing schools' administrators, nursing educators and nursing managers and nursing staff in clinical settings. Nursing schools are encouraged to review nursing curricula, assignments and workload, prepare and equip students with the needed knowledge and skills prior clinical rotation. It is recommended that faculty use simulated scenarios such as high‐fidelity simulators to help familiarize students with clinical conditions prior to their first clinical experience. Junior students need special support and creating a motivating clinical environment represent a priority for them. Moreover, there is a need to offer nursing students frequent workshops on stress management and problem solving to help alleviate academic stress, along with it a concurrent evaluation of desired outcomes. Students with limited financial resources need special attention as well. Students from current study sample had additional stress as a result of financial burden and other commitments towards their work, lives and family. There is a need to provide financial aid for those disadvantaged students to overcome the financial obstacles they face.

## CONFLICT OF INTEREST

The authors declare that there is no conflict of interest that could be perceived as prejudicing the impartiality of the research reported.

## Data Availability

The data are not publicly available due to restrictions, for example their containing information that could compromise the privacy of research participants.
